# Dietary Patterns and Determinants of Pregnant and Lactating Women From Marginalized Communities in India: A Community-Based Cross-Sectional Study

**DOI:** 10.3389/fnut.2020.595170

**Published:** 2020-11-13

**Authors:** Shantanu Sharma, Faiyaz Akhtar, Rajesh Kumar Singh, Sunil Mehra

**Affiliations:** ^1^Department of Clinical Sciences, Skåne University Hospital, Lund University, Malmö, Sweden; ^2^Reproductive, Maternal, Newborn, Child and Adolescent Health Division, MAMTA Health Institute for Mother and Child, New Delhi, India

**Keywords:** diet survey, diet quality, factor analysis, health service, nutrition assessment, pregnancy

## Abstract

**Objective:** Dietary pattern analysis has emerged as a balanced and realistic approach that reflects how the food is consumed in real life. However, previous studies have overlooked the two important phases in women's life, pregnancy, and lactation. We aimed to explore dietary patterns and their determinants among pregnant and lactating women from marginalized families in rural areas and urban slums of India.

**Methods:** It was a community-based cross-sectional study conducted across four districts of India, one from each region (North, West, East, and South). We used a structured questionnaire to collect data on socio-demographic characteristics and access to nutrition services. The dietary data were collected using a qualitative food frequency questionnaire having 204 food items, which were clubbed into 16 major food groups. The principal component analysis method was employed to identify dietary patterns (prefixed at 4). We used multinomial logistic regression to explore associations of socio-demographic and access to nutrition services' variables with identified dietary patterns.

**Results:** The data of 476 pregnant and 446 lactating women were assessed. Four patterns explained for 54% of the variation in their food intake. The patterns identified were low-mixed vegetarian (19%), non-vegetarian (15%), high-mixed vegetarian (11%), and calorie-rich (9%). The low-mixed diet was rich in rice, roots and tubers, green leafy vegetables, and other vegetables. The non-vegetarian diet was characterized by high loadings for nuts or seeds, chicken, meat or fish, eggs, beverages (milk-based), and snacks. The high-mixed vegetarian diet was rich in cereals other than rice and wheat, pulses, and fruits. The calorie-rich diet had high factor loadings for wheat, butter and oil, sweets, and milk and milk products. Hindus and women who lived in rural areas had higher odds of consuming a low-mixed vegetarian diet and lower odds of a high-mixed vegetarian diet. Working women and those who received nutrition advice during pregnancy or lactation had 2–3 times higher odds of consuming a high-mixed vegetarian diet.

**Conclusions:** A high prevalence of a low-mixed vegetarian diet among women can have adverse pregnancy and birth outcomes. Healthy dietary patterns during pregnancy and lactation are required to meet the increased micro- and macronutrient requirements for improved maternal and child health.

## Introduction

In the move toward prioritizing “1,000 days,” adequate maternal nutrition is paramount in improving the nutrition status of a child and reducing the risk of adverse fetal outcomes such as intrauterine growth retardation, pre-term birth, and low birth weight (1). Micronutrient deficiencies and inadequate dietary intake in women of reproductive age have transgenerational effects by altering the developmental trajectory of the fetus (2). Increased vulnerabilities and prevalence of malnutrition among women of reproductive age arise from a mismatch between enhanced demands for protein, energy, and micronutrients and improper replenishment during pregnancy and lactation (3). Pregnancy, in its entire duration, consumes about 60,000 kcal over and above the normal metabolic requirements (4). There is empirical evidence on the suboptimal consumption of micronutrients, inadequate weight gains, and high prevalence of nutritional anemia among pregnant women from Low Middle-Income Countries (5).

India, with an average rate of ~24 million births per year (6), ranks as the second most populated country in the world and is also the bottom-most Asian country in the mother index rankings (7). The National Family Health Survey (NFHS) round four documented that 50–57% of pregnant women and lactating mothers were anemic in India, and their diet lacked food rich in micronutrients (8). It is imperative to understand that the determinants of maternal malnutrition are multifactorial, including poverty, ignorance, food insecurity, and inappropriate food distribution, which adds socio-economic angle to this public health problem (9). Together with the inadequate dietary intake, and inequitable household food distribution, food taboos, misconceptions, recurrent infections, and poor care constitute significant contributors to undernutrition among pregnant women and lactating mothers (10).

Socio-economic and demographic factors have a substantial effect on nutritional status and the food intake of women, especially pregnant and lactating women in the family. Previous literature has highlighted the influence of factors such as women's education status, family size, and community to which a woman belongs to their nutritional status and intake (11, 12). Considering this as a significant public health issue, the national government in India has taken up several initiatives to address various dimensions of maternal nutrition (1). Integrated Child Development Service Scheme (ICDS), one of India's flagship programs, has a positive impact on mothers and children (13, 14). *Anganwadi* centers (ICDS centers, rural maternal and child care centers), are places where pregnant and lactating women receive supplementary food, health and nutrition education, and other services like iron folic tablets (13).

There is a need for a new and more aggressive focus on coupling effective nutrition-specific interventions (i.e., those that address the immediate determinants of nutrition) with nutrition-sensitive programs that address the underlying causes of maternal undernutrition (15). In that regard, nutritional surveillance becomes indispensable for gathering, processing, and analyzing nutritional indicators to monitor such interventions. Conventionally, nutrition surveillance has focused on “one nutrient or food” approach. On the contrary, the dietary pattern analysis has emerged as a balanced and realistic approach that reflects how the food is consumed in real life (16). Understanding the dietary patterns using the food frequency questionnaire (FFQ) technique is a simple, cost-effective, and time-efficient dietary assessment method suitable for community-based nutritional surveillance on a large scale (17).

However, there is limited availability of data on dietary patterns in India. Further, of the available data, there are methodological limitations, such as orthogonal rotation of the factors to allocate individuals to patterns, thereby making it difficult to ascertain the validity of the patterns (18). The previous studies had examined the dietary patterns of adult females or adult males or children without emphasizing the two important phases in the life of women, pregnancy, and lactation (18). There is a considerable body of evidence suggesting changes in the dietary habits or patterns of women during pregnancy or lactation due to traditional beliefs, cultural reasons, personal values, or preferences (19–21). Therefore, the present study explored dietary patterns and their determinants among pregnant women and lactating mothers from marginalized families in rural areas and urban slums of India. Our dietary pattern analysis goal was to summarize a large number of correlated dietary variables obtained through a qualitative FFQ into fewer independent components.

## Materials and Methods

### Study Sites, Sampling, and Sample Size

The study was conducted across four states of India as a part of project *JAGRITI* (means awakening), a community-based intervention, to generate evidence on the dietary practices of the target groups. The technical details of the project, including strategy and intervention, are available elsewhere (22). One state from each region of modern India—Delhi, Karnataka, Bihar, and Rajasthan from the North, South, East and West regions, respectively, were selected. One district per state and one block per district were randomly selected for the survey from the Census using a random number table. Further, the top 10 villages/wards with the highest proportion of marginalized populations, including scheduled castes, tribes, and other marginalized classes in each block, were selected. For each village/ward, the number of households selected was proportional to their ratio of a marginalized population. The first household was chosen randomly in each area, and subsequent households were covered based on the fraction obtained during systematic random sampling. Using a 22% prevalence of malnutrition among women from marginalized populations (8), at 95% confidence level, 5% absolute error, design effect of 1.5, and 5% drop rate, the sample size was calculated at 433 for each group. Only women residing in the area for the past 1-year or more were interviewed. The study employed a cross-sectional quantitative study design.

### Ethical Clearance

Data were collected between October and December 2016. The Institutional ethical review board granted approval for the study (MERB/Sep.2016/003). The objectives of the study were explained to the participants in their local language (Hindi/kannad), and written informed consent was obtained. A total of 476 pregnant women (between 4th and 9th months of pregnancy) and 446 lactating mothers (having a child between 0 and 2 years of age) from all the four blocks were interviewed.

### Study Tool

The qualitative FFQ was interviewer-based and administered in the local language by eight trained research assistants. The FFQ was developed and evaluated for test-retest reproducibility using kappa statistics and spearman correlations (data not shown). The FFQ of 204 food items was used to assess the usual daily intake of foods and nutrients. Each food item has a value for 57 nutrients, including energy, proteins, vitamins, and minerals. The questionnaire had 11 possible responses, ranging from “never or occasional, once in 3 months, once a month, to once a week or once daily, or twice or more daily.” They were converted into daily equivalents. This could be understood from an example if a woman ate rice twice daily, then the daily equivalent was 2, and if a woman ate snacks once in a week, the daily equivalent was 0.14. Portion sizes were not assessed in the FFQ, so grams per day of the foods consumed were not available. Hence, dietary intake frequencies were used to determine dietary patterns. The 204 food items were clubbed into 16 major food groups according to their nutritional content and the 2011 Dietary Guidelines for Indians (23). The 16 food groups included cereals (wheat, rice, and other cereals such as maize, millet, or corn), roots and tubers, pulses, milk (milk products), non-vegetarian food items (meat, chicken, and fish), eggs, fruits, green leafy vegetables, nuts and seeds, snacks, sweets, beverages, butter or oil, and other vegetables. We analyzed rice and wheat separately because wheat is a staple cereal in Northern and Western India, and rice is a staple cereal in Eastern and Southern India. Cronbach's α was used to measure the internal consistency of the tool with socio-demographic and access to nutrition services' questions, the value of which was 0.7.

All the variables pertaining to pregnant and lactating mothers were divided into two broad categories, namely socio-demographic and access to health services. The following variables were included in the “socio-demographic” category: religion (Hindu, Muslim, Jain, and others), type of family (nuclear or joint), education and occupation status of women, social class (scheduled caste or tribe or other marginalized classes or non-marginalized class), residential area type (a rural area or an urban slum), socio-economic status of the family, age, and gestational age of women. Maternal education was categorized as illiteracy or educated until primary, secondary or high school, and university level or above. Women's occupation was grouped into five categories, namely unskilled labor (laborers, farmers, maids, servants, gatekeepers, cleaner, helper, sweeper), semi-skilled work (artisans, taxi drivers, waiters), skilled job (service, small or big business, technicians, electricians, tailors, cooks), housewives, and unemployed. The socio-economic status of women was assessed using the modified *Kuppuswamy* scale. The modified *Kuppuswamy* scale is based on three parameters- education and occupation of the head of the household, and monthly family income (24). The scale is grouped into five categories, namely lower, upper-lower, lower-middle, upper-middle, and upper categories. The following variables were included in the “access to services” by women: receiving nutrition-related advice during pregnancy or lactation and supplementary food from *Anganwadi* centers.

### Statistical Analysis

The principal component analysis (PCA) method was used to identify dietary patterns (25). We prefixed the number of factors to four meaningful patterns. In the process of identifying four key dietary patterns from a large number of factor solutions in PCA, we adopted the following criteria: (1) Factors with eigenvalues >1.0 were included (2) orthogonal (Varimax) rotation of the identified factor structure was done (3) In each principal component factor, food groups with factors loadings ≥0.30 or ≤-0.30 were included (4) numbering and labeling of the factors were done. The Kaiser-Meyer-Olkin (KMO) measure reached the acceptable limit of 0.6, and Bartlett's test of sphericity was significant (*p* < 0.001), meaning thereby that the data were suitable for factor analysis.

Data were expressed as frequency and percentages for the categorical variables and mean (Standard Deviation, SD) or median (Interquartile Range, IQR) for continuous variables. The dietary pattern scores were dichotomized into tertiles. The unadjusted and adjusted multinomial logistic regression was employed to investigate the relationship of the four dietary patterns scores with determinants using main-effects model. Multi-collinearity among the predictors was assessed using the variance inflation factor, and Eigen values of the variables in the collinearity diagnostic table. The multinomial model of regression was fitted with socio-demographic variables and access to services factors in the same model (age was used as the covariate). For the regression analysis, the five categories of the *Kuppuswamy* scale were merged to give two groups: lower and upper-lower as one, and the other included the rest. Similarly, women's occupation status was regrouped as working or not working for regression analysis. Odds ratio and 95% confidence intervals were used to depict the strength and precision of associations. All the quantitative variables were normally distributed. Data analyses were performed with the IBM SPSS Statistics for Windows version 25.0 (IBM Corp., Armonk, N.Y., USA).

## Results

**Figure 1:**
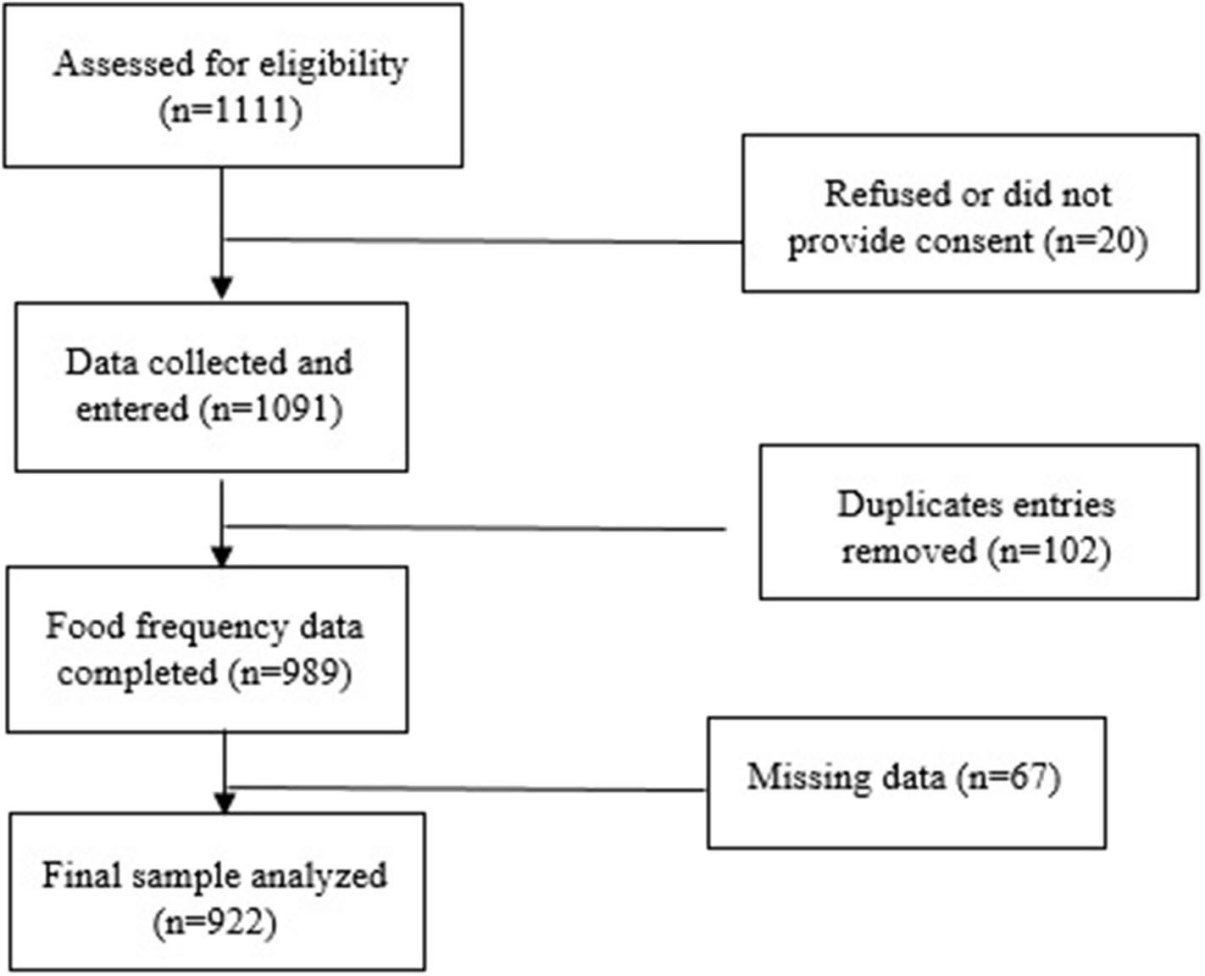
Flowchart of participant progression through the dietary survey across 4 districts of India.

Of the 1,111 women assessed for eligibility, 1,091 women provided consent for the study, and their data were collected (Figure 1). Hundred and two duplicate entries and 67 entries with missing data were removed. Finally, a total of 922 participants (476 pregnant women and 446 lactating mothers) were included. The mean age of pregnant women and lactating mothers were similar, 23.6 (± 3.4), and 24.8 (± 3.6) years, respectively (Table 1). The mean gestational age of the pregnant women at the time of the interview was 6.3 (± 1.6) months. Nearly 80% of all the participants belonged to scheduled caste or tribes and other marginalized classes. Nearly 30% of both pregnant and lactating women had obtained education till the primary level or below. The majority of the women (>92%) were housewives (Table 1). Around 38–40% of the participants did not receive nutritional advice during their respective phases (pregnancy or lactation).

**Table 1:** Participant's socio-demographic characteristics by groups (*n* = 922).

	**Pregnant women (*n* = 476) *N* (%)**	**Lactating women (*n* = 446) *N* (%)**
Age in years (Mean ± SD)	23.6 (± 3.4)	24.8 (± 3.6)
Age of gestation at the time of interview in months (Mean ± SD)	6.3 ± 1.6	—-
**Socio-demographic variables**		
**Religion**		
Hindu	389 (81.7)	349 (78.3)
Muslim	36 (7.6)	38 (8.5)
Jains	32 (6.7)	30 (6.7)
Others^†^	19 (4.0)	29 (6.5)
**Social class**		
Scheduled caste or tribe	246 (51.7)	219 (49.1)
Other marginalized classes	131 (27.5)	129 (28.9)
Non-marginalized classes	99 (20.8)	98 (22.0)
**Type of family**		
Nuclear	223 (46.8)	234 (52.5)
Joint	253 (53.2)	212 (47.5)
**Educational status of women**		
Illiterate and primary education	145 (30.5)	137 (30.7)
Secondary and high school education (5th−12th)	290 (60.9)	268 (60.1)
University degree and above	41 (8.6)	41 (9.2)
Occupational status of women^*^		
Unskilled labor	20 (4.2)	20 (4.5)
Semiskilled work	6 (1.3)	–
Skilled job	2 (0.4)	5 (1.1)
Housewife	440 (92.4)	413 (92.6)
Unemployed	8 (1.7)	8 (1.8)
**Residential area**		
Rural	244 (51.3)	218 (48.9)
Urban slums	232 (48.7)	228 (51.1)
**Socioeconomic status of the family**		
Lower	14 (2.9)	21 (4.7)
Upper-lower	428 (89.9)	319 (71.5)
Lower-middle	23 (4.8)	91 (20.4)
Upper-middle	11 (2.3)	15 (3.4)
**Access to nutrition services**		
**Nutrition-related advice received during pregnancy or lactation**		
Yes	296 (62.2)	264 (59.2)
No	180 (37.8)	182 (40.8)
**Women receiving supplementary nutrition from** ***Anganwadi*** **Center**		
Not received	227 (47.7)	214 (48.0)
Received	249 (52.30)	232 (52.0)

*INR, Indian Rupees; SD, Standard Deviation*.

* Occupations: Unskilled work included maids, servants, gatekeepers, cleaner, helper, sweeper, farmer, etc.; semiskilled work include drivers, waiters, etc.; skilled work includes technicians, electricians, tailors, cooks, etc.;

† *Others include Sikhs and Christians*.

### Principal Component Analysis of Dietary Patterns

The four principal components explained the largest proportions of variance in the dietary intake among women and had eigenvalues more than 1.0. These components were retained as four dietary patterns among pregnant and lactating women (Table 2). Together, these four patterns explained 54% (18.9, 15, 10.9, and 8.9%, respectively) of the variation in food intake. Factor 1 was characterized by high factor loadings for rice, roots and tubers, green leafy vegetables, and other vegetables. Factor 2 was characterized by high loadings for nuts or seeds, chicken, meat or fish, eggs, beverages (milk-based), and snacks. Factor 3 was characterized by high loadings for cereals other than rice and wheat, pulses, and fruits. In addition, factor 3 had >0.3-factor loadings for milk and milk products and nuts and seeds. Factor 4 was characterized by high factor loadings for wheat, butter and oil, sweets, and milk and milk products.

**Table 2:** Factor loadings for food groups that loaded highly (|>0.30|) in Varimax rotated principal components for women.

**Food groups**	**LMV**	**NV**	**HMV**	**CR**
Eigen value	3.02	2.41	1.75	1.43
% variance explained	18.91	15.08	10.94	8.97
Wheat	−0.34	0.17	−0.65	**0.35**
Rice	**0.67**	0.27	0.22	−0.25
Other cereals	−0.05	−0.05	**0.42**	−0.02
Roots and tubers	**0.77**	−0.14	−0.27	0.00
Pulses or lentils	0.04	0.24	**0.73**	0.00
Green leafy vegetables	**0.88**	−0.03	−0.02	0.04
Other vegetables	**0.88**	0.12	0.09	0.02
Fruits	−0.18	**0.31**	**0.47**	0.23
Chicken or meat or fish	0.09	**0.55**	0.19	−0.32
Eggs	0.18	**0.66**	0.09	0.05
Milk and milk products	0.02	−0.24	**0.38**	**0.63**
Nuts and seeds	0.02	**0.41**	**0.31**	0.19
Butter and oil	−0.10	0.06	−0.10	**0.76**
Beverages	−0.16	**0.58**	0.01	−0.06
Sweets	0.09	**0.40**	−0.09	**0.52**
Snacks	0.03	**0.68**	−0.16	0.19

*CR, Calorie-Rich; HMV, High-mixed vegetarian; LMV, Low-mixed vegetarian; NV, Non-vegetarian Positive factor loadings >0.3 have been highlighted in bold*.

The first dietary pattern was named low-mixed vegetarian, rich in only vegetables, roots, and rice. The second and third patterns (Factor 2 and 3, respectively) were named non-vegetarian and high-mixed vegetarian dietary patterns. The fourth pattern, high in sweets and oil, was named as a calorie-rich dietary pattern.

### Associations Between Dietary Patterns and Women's Socio-Demographic Characteristics and Access to Nutrition Services

As shown in Tables 3, 4, the low-mixed vegetarian dietary pattern was common among women who were Hindus, lived in rural areas, belonged to marginalized classes, and not received supplementary nutrition than their counterparts. Women who had the highest grades of education, belonged to marginalized classes, were working, and received nutrition advice had higher odds of consuming a high-mixed vegetarian diet. The non-vegetarian diet was uncommon among marginalized classes. On the contrary, women who were Hindus, lived in urban slums, and did not receive supplementary nutrition had higher odds of consuming a non-vegetarian diet. In the unadjusted analysis, women from nuclear families and high socio-economic strata also had higher odds of consuming a non-vegetarian diet, but not in the adjusted analysis. The calorie-rich pattern was common among women who lived in rural areas. Although increasing age was associated with non-vegetarian and high-mixed vegetarian dietary patterns in the unadjusted analysis, the associations turned insignificant after adjustments for other variables.

**Table 3:** Unadjusted multinomial logistic regression for the dietary patterns' associations with socio-demographic variables and access to nutrition services among women (*n* = 922).

**Variables**	**Low-mixed vegetarian OR (95%CI)**		**Non-vegetarian OR (95%CI)**		**High-mixed vegetarian OR (95%CI)**		**Calorie-rich OR (95%CI)**	
	**Highest**	**Middle**	**Highest**	**Middle**	**Highest**	**Middle**	**Highest**	**Middle**
Age	0.97 (0.93, 1.02)	1.01 (0.96, 1.05)	1.05 (1.01, 1.11)^*^	1.04 (0.99, 1.08)	**1.01 (0.96, 1.05)**	0.98 (0.94, 1.03)	0.98 (0.94, 1.03)	0.99 (0.95, 1.04)
**Religion**								
Hindu Non-Hindu	2.35 (1.51, 3.66)^***^ *Reference*	0.91 (0.62, 1.32) *Reference*	1.39 (0.95, 2.06) *Reference*	1.72 (1.15, 2.57)^**^ *Reference*	0.36 (0.23, 0.54)^***^ *Reference*	0.81 (0.51, 1.27) *Reference*	0.84 (0.56, 1.27) *Reference*	0.73 (0.48, 1.09) *Reference*
**Social class**								
Scheduled caste or tribe Other marginalized Non-marginalized	3.15 (2.01, 4.94)^***^ 4.27 (2.61, 6.98)^***^ *Reference*	1.66 (1.12, 2.46)^*^ 1.67 (1.06, 2.63)^*^ *Reference*	0.13 (0.07, 0.21)^***^ 0.35 (0.19, 0.58)^***^ *Reference*	0.17 (0.10, 0.29)^***^ 0.34 (0.19, 0.61)^***^ *Reference*	18.86 (9.48, 37.50)^***^ 14.83 (7.26, 30.28)^***^ *Reference*	1.96 (1.34, 2.88)^**^ 1.68 (1.09, 2.58)^*^ *Reference*	1.45 (0.97, 2.15) 1.27 (0.81, 1.97) *Reference*	2.53 (1.64, 3.89)^***^ 2.07 (1.29, 3.33)^**^ *Reference*
**Type of family**								
Nuclear Joint	0.79 (0.57, 1.09) *Reference*	0.96 (0.70, 1.33) *Reference*	2.93 (2.10, 4.09)^***^ *Reference*	2.24 (1.61, 3.11)^***^ *Reference*	0.62 (0.45, 0.86)^**^ *Reference*	0.78 (0.57, 1.08) *Reference*	0.74 (0.53, 1.02) *Reference*	0.61 (0.44, 0.84)^**^ *Reference*
**Education status of women**								
Illiterate and primary school Secondary and high school University degree and above	1.79 (0.98, 3.25) 0.85 (0.48, 1.50) *Reference*	0.90 (0.48, 1.69) 1.28 (0.73, 2.27) *Reference*	0.41 (0.22, 0.77)^**^ 0.61 (0.34, 1.10) *Reference*	0.79 (0.41, 1.51) 0.79 (0.42, 1.47) *Reference*	0.40 (0.21, 0.76)^**^ 1.18 (0.66, 2.10) *Reference*	1.05 (0.57, 1.91) 0.83 (0.46, 1.50) *Reference*	0.64 (0.35, 1.18) 0.93 (0.52, 1.67) *Reference*	0.57 (0.31, 1.07) 1.08 (0.60, 1.95) *Reference*
**Occupation**								
Working Not working	0.85 (0.45, 1.60) *Reference*	0.85 (0.45, 1.62) *Reference*	0.40 (0.20, 0.80)^*^ *Reference*	0.69 (0.38, 1.25) *Reference*	2.18 (1.01, 4.71)^*^ *Reference*	3.10 (1.48, 6.49)^**^ *Reference*	1.17 (0.62, 2.21) *Reference*	0.99 (0.51, 1.92) *Reference*
**Residential area**								
Rural Urban slums	6.93 (4.79, 10.03)^***^ *Reference*	0.67 (0.47, 0.94)^*^ *Reference*	0.02 (0.01, 0.03)^***^ *Reference*	0.09 (0.06, 0.14)^***^ *Reference*	1.26 (0.91, 1.75) *Reference*	3.26 (2.33, 4.55)^***^ *Reference*	1.63 (1.18, 2.25)^**^ *Reference*	1.18 (0.85, 1.63) *Reference*
**Socio-economic class**								
Lower and upper-lower Middle and above	1.07 (0.70, 1.65) *Reference*	1.44 (0.91, 2.26) *Reference*	0.38 (0.23, 0.61)^***^ *Reference*	0.62 (0.38, 1.03) *Reference*	2.47 (1.52, 4.03)^***^ *Reference*	1.14 (0.75, 1.72) *Reference*	1.19 (0.77, 1.85) *Reference*	1.18 (0.76, 1.84) *Reference*
**Supplementary food received**								
No Yes	1.97 (1.42, 2.72)^***^ *Reference*	1.04 (0.75, 1.44) *Reference*	3.64 (2.59, 5.12)^***^ *Reference*	3.71 (2.64, 5.22)^***^ *Reference*	0.96 (0.69, 1.32) *Reference*	0.92 (0.67, 1.27) *Reference*	0.79 (0.57, 1.09) *Reference*	0.64 (0.46, 0.08)^**^ *Reference*
**Nutritional advice received**								
Yes No	3.39 (2.38, 4.84)^***^ *Reference*	1.03 (0.75, 1.42) *Reference*	0.72 (0.52, 1.01) *Reference*	0.73 (0.52, 1.01) *Reference*	1.55 (1.12, 2.15)^**^ *Reference*	1.87 (1.34, 2.61)^***^ *Reference*	1.07 (0.77, 1.50) *Reference*	0.84 (0.60, 1.16) *Reference*

*Lowest tertile was the reference category*.

*OR, Unadjusted Odds Ratio; 95%CI, 95% Confidence Interval*.

* P < 0.05;

** p < 0.01;

*** *p < 0.001*.

**Table 4:** Adjusted multinomial logistic regression for the dietary patterns' associations with socio-demographic variables and access to nutrition services among women (*n* = 922).

**Variables**	**Low-mixed vegetarian aOR (95%CI)**		**Non-vegetarian aOR (95%CI)**		**High-mixed vegetarian aOR (95%CI)**		**Calorie-rich aOR (95%CI)**	
	**Highest**	**Middle**	**Highest**	**Middle**	**Highest**	**Middle**	**Highest**	**Middle**
Age	0.99 (0.94, 1.05)	1.01 (0.96, 1.05)	0.97 (0.91, 1.04)	0.99 (0.93, 1.05)	1.03 (0.98, 1.09)	0.99 (0.95, 1.04)	1.00 (0.95, 1.05)	1.01 (0.96, 1.06)
**Religion**								
Hindu Non-hindu	1.71 (1.04, 2.80)^*^ *Reference*	0.86 (0.57, 1.30) *Reference*	3.19 (1.74, 5.83)^***^ *Reference*	2.84 (1.64, 4.92)^***^ *Reference*	0.23 (0.14, 0.38)^***^ *Reference*	0.62 (0.38, 1.01) *Reference*	0.83 (0.54, 1.28) *Reference*	0.76 (0.49, 1.17) *Reference*
**Social class**								
Scheduled caste or tribe Other marginalized Non-marginalized	1.42 (0.79, 2.57) 2.50 (1.42, 4.70)^**^ *Reference*	**2.22 (1.41, 3.50**)^**^ 1.76 (1.08, 2.85)^*^ *Reference*	0.93 (0.47, 1.84) 2.24 (1.10, 4.54)^*^ *Reference*	0.76 (0.40, 1.46) 1.45 (0.73, 2.86) *Reference*	34.59 (16.04, 74.61)^***^ 15.63 (7.27, 33.59)^***^ *Reference*	0.95 (0.58, 1.56) 0.90 (0.55, 1.49) *Reference*	1.06 (0.67, 1.69) 0.94 (0.58, 1.53) *Reference*	2.54 (1.55, 4.17)^***^ 1.90 (1.14, 3.16)^*^ *Reference*
**Type of family**								
Nuclear Joint	1.51 (1.00, 2.29) *Reference*	0.86 (0.60, 1.22) *Reference*	1.29 (0.81, 2.05) *Reference*	1.29 (0.85, 1.95) *Reference*	0.74 (0.49, 1.11) *Reference*	1.16 (0.80, 1.67) *Reference*	0.94 (0.66, 1.33) *Reference*	0.67 (0.47, 0.97)^*^ *Reference*
**Education status of women**								
Illiterate and primary school Secondary and high school University degree and above	1.70 (0.70, 3.65) 1.01 (0.49, 2.07) *Reference*	0.90 (0.45, 1.77) 1.15 (0.62, 2.11) *Reference*	1.32 (0.55, 3.18) 0.85 (0.38, 1.91) *Reference*	1.62 (0.71, 3.69) 1.03 (0.48, 2.23) *Reference*	0.22 (0.10, 0.48)^***^ 0.69 (0.34, 1.41) *Reference*	0.73 (0.36,1.45) 0.79 (0.41,1.50) *Reference*	0.50 (0.26, 0.98)^*^ 0.83 (0.45, 1.53) *Reference*	0.51 (0.26, 1.01) 0.93 (0.49, 1.75) *Reference*
**Occupation**								
Working Not working	0.64 (0.31, 1.31) *Reference*	0.96 (0.49, 1.86) *Reference*	0.71 (0.28, 1.78) *Reference*	1.00 (0.49, 2.06) *Reference*	2.62 (1.13, 6.05)^*^ *Reference*	2.63 (1.22, 5.67)^*^ *Reference*	0.98 (0.49, 1.93) *Reference*	1.24 (0.64, 2.41) *Reference*
**Residential area**								
Rural Urban slums	9.67 (5.80, 16.11)^***^ *Reference*	0.44 (0.28, 0.69)^***^ *Reference*	0.02 (0.01, 0.03)^***^ *Reference*	0.10 (0.06, 0.18)^***^ *Reference*	0.53 (0.34, 0.82)^**^ *Reference*	3.21 (2.14, 5.03)^***^ *Reference*	1.64 (1.09, 2.48)^*^ *Reference*	0.84 (0.56, 1.26) *Reference*
**Socio-economic class**								
Lower and upper-lower Middle and above	0.66 (0.38, 1.14) *Reference*	1.39 (0.85, 2.25) *Reference*	0.64 (0.33, 1.21) *Reference*	0.96 (0.52, 1.78) *Reference*	2.04 (1.14, 3.68)^*^ *Reference*	0.90 (0.57, 1.45) *Reference*	1.22 (0.75, 1.97) *Reference*	1.10 (0.68, 1.78) *Reference*
**Supplementary food received**								
No Yes	3.26 (2.06, 5.15)^***^ *Reference*	0.92 (0.61, 1.39) *Reference*	3.00 (1.80, 5.00)^***^ *Reference*	3.95 (2.51, 6.22)^***^ *Reference*	1.06 (0.68, 1.66) *Reference*	0.92 (0.62, 1.37) *Reference*	0.83 (0.57, 1.23) *Reference*	0.71 (0.47, 1.05) *Reference*
**Nutritional advice received**								
Yes No	1.31 (0.84, 2.04) *Reference*	1.12 (0.76, 1.65) *Reference*	0.84 (0.49, 1.45) *Reference*	0.51 (0.31, 0.83)^**^ *Reference*	1.90 (1.21, 2.99)^**^ *Reference*	1.59 (1.05, 2.40)^*^ *Reference*	1.10 (0.74, 1.64) *Reference*	1.01 (0.68, 1.52) *Reference*

*Lowest value category was the reference category; PsuedoR^2^ for the model of the first factor as a dependent variable was 0.31 (Cox Snell) and 0.35 (Nagelkerke); R^2^ for the model of the second factor as a dependent variable was 0.41 (Cox Snell) and 0.46 (Nagelkerke); R^2^ for the model of the third factor as a dependent variable was 0.30(Cox Snell) and 0.34 (Nagalkerke); and R^2^ for the model of the fourth factor as a dependent variable was 0.06 (Cox Snell) and 0.34 (0.07)*.

*aOR, Adjusted Odds Ratio; 95%CI, 95% Confidence Interval*.

* P < 0.05;

** p < 0.01;

*** *p < 0.001*.

## Discussion

In the present study, four dietary patterns for pregnant and lactating women were identified. The results of the study highlight the key dietary patterns and their associations with different socio-demographic characteristics and access to nutrition services in pregnant and lactating women.

Illiteracy or <5 years of schooling is prevalent among Indian women (20–30%), more common among women from marginalized classes than non-marginalized women (8). Female labor participation is meager (<30%), and lower among women from urban areas than rural villages (26). Our findings are congruent with these socio-demographic findings of Indian women. Most of the participants belonged to lower or upper-lower socio-economic strata in our study, which doesn't reflect the true population status as described in the national level report (8). A higher representation from the upper-lower socio-economic strata arose out of the objectives of the study to focus on socially and economically marginalized populations. This unequal distribution of study populations may affect the dietary patterns emerging out of the data and make findings less generalizable to other areas with less proportion of this segment of the population.

In our study population, nearly half of the pregnant and lactating women received supplementary food, which is almost similar to the NFHS-4 findings. Around 60–62% of pregnant or lactating women in our study received nutrition-related advice similar to the proportion that was reported in the NFHS-4 findings (8).

Notably, a low-mixed vegetarian diet without fruits and pulses was the predominant pattern among women. Predominantly, vegetables and cereal-based diets among pregnant women in rural areas have been reported in other studies from India (27–30). Green leafy vegetables are a good source of iron but have low bioavailability compared to animal sources. Indians mostly have a vegetarian diet with little meat consumption and suffer from a high prevalence of anemia (31). Besides, evidence suggests that women in India have poor quality diets with infrequent consumption of animal source foods and micronutrient-rich fruits and vegetables (32). A recent systematic review assessing published and gray literature to identify common dietary patterns in India reported similar findings. The review revealed that most dietary patterns were vegetarian with a predominance of fruit, vegetables, and pulses, and cereals. In addition, dietary patterns based on high-fat, high-sugar foods, and more meat were also identified (18).

The pulses- and fruits-rich high-mixed vegetarian pattern was a distinct pattern in the present study and did not aggregate with the common vegetarian pattern. This doesn't concur with the previous studies citing the vegetarian diet as the major dietary pattern rich in fruits, vegetables, and pulses (18). This difference in results might be because of the higher representation of the study population from upper-lower socio-economic strata and marginalized classes in our study. In the systematic review of eight studies assessing 11 separate models of dietary patterns in India, most of the included studies did not either assess socio-economic strata or did not use a standard scale like *Kuppuswamy* and look at their associations with dietary patterns (18).

The additional dietary patterns, non-vegetarian and calorie-rich, have also been suggested in other studies (18, 33). The calorie-rich dietary pattern with high milk and milk product consumption is an age-old tradition among Indian women where they are fed more milk with the notion that it would enhance their breast milk production. The study, based on the GUSTO birth cohort, also reported similar results (34). The study mentioned that the consumption of leafy vegetables and milk-based drinks increased post-pregnancy among Indian participants compared to the pre-pregnancy period from 47.1 to 73.4% (*p* < 0.001).

Based on NFHS-2 data, the analysis of the dietary patterns among adult women revealed that a low-mixed diet (predominantly vegetarian) was more common among Hindus in rural areas with high standards of living (35). This is congruent with our study results. Besides, our data investigations showed that women who belonged to low socio-economic strata were less likely to consume a diet rich in non-vegetarian foods. This suggests how affordability to buy costlier non-vegetarian food items shapes the dietary patterns (35). This is equally important to understand that the consumption of non-vegetarian food items decreases/is low during pregnancy or lactation (36). As a result, the dietary patterns of pregnant and lactating women are different and difficult to compare with the studies that have assessed non-pregnant women primarily. There is a mix of evidence on the association of household income with dietary consumption and patterns (37, 38). However, the present endeavor observed lower odds of consuming non-vegetarian food and higher odds of consuming a high-mixed vegetarian diet among women from lower or upper-lower-wealth quintiles. This paradox could be explained by increased access to *Anganwadi centers* by women from lower socio-economic strata. In recent times, these centers are adopted and run by various governmental or non-governmental organizations providing nutritious meals, including fruits, nuts-embedded sweets, and energy-dense foods. The government of India has made strenuous efforts to upgrade these centers across states (39).

The receipt of supplementary nutrition from *Anganwadi* centers was found to be associated with decreased consumption of low-mixed or non-vegetarian diet among pregnant and lactating women in our study. ICDS centers are supposed to provide nutrition education to women besides supplementary food and hence, should promote the consumption of a diverse diet (13). Lower odds of non-vegetarian food intake can be understood from the lack of effective implementation of the policy to distribute eggs in *Anganwadi* centers (40, 41) and a lack of access to *Anganwadi* centres by women who have a higher consumption of non-vegetarian foods (women from higher socio-economic strata).

The result that the women who received nutrition advice and were working were more likely to consume a diet rich in pulses or fruits (high-mixed) is highly convincing. Congruent to our findings, another study found in a randomized controlled intervention trial that pregnant and lactating women who received nutrition-related counseling had a higher likelihood of consuming a diverse diet (42). This spotlights the fact that women's empowerment (employment) is a key driver for attaining maternal and child health and nutritional goals (43).

Calorie-rich dietary pattern was more common among women residing in rural areas or women with higher grades of education. The distinct pattern of the intake of milk or milk products, sweets, and butter or oil among women may be due to socio-cultural norms, food preferences or the easy availability of such items or their increased advertisement through mass media (44). The dietary patterns of young women and men have changed due to the greater influence of western lifestyle with increased intake of readily available junk foods. Studies found an increase in the per capita consumption of processed food in India with increasing income and urbanization (45). These ultra-processed foods rich in sugar and salt and poor in iron, calcium, and dietary fiber affect dietary patterns negatively (46).

Since the majority of the population in the study belonged to marginalized classes, it is surprising to see higher odds of a high-mixed vegetarian diet among them non-marginalized ones. The national survey suggested that the consumption of most of the food items, including pulses, fruits, milk, egg, and meat, is less common among marginalized classes than non-marginalized ones (8). Furthermore, the survey suggested that the consumption of eggs, fruits, fish, and meat is nearly half as prevalent as vegetables and/or pulses. On the contrary, the consumption of fried food items is equally as common as the consumption of fruits (8). This underpins the need for a comprehensive outlook on behavioral interventions that improve the dietary habits of pregnant and lactating women amidst considerable socio-cultural and economic influences.

### Importance of the Study

The study highlights the need to assess the dietary patterns and their determinants of women during pregnancy and lactation. Other studies reported that unhealthy dietary patterns or low-mixed diets (lack of non-vegetarian diet or fruits or pulses) are associated with adverse pregnancy and birth outcomes like gestational hypertension, pre-term delivery, gestational diabetes mellitus, etc. (47, 48). Maternal diet during pregnancy has a significant impact on the levels of fatty acids in erythrocytes and breast milk. Fatty acids, such as arachidonic acid and docosahexaenoic acid, have a relevant role in the different metabolic and physiological processes during embryonic and fetal development and the first years of life (49).

### Limitations and Strengths

The study has several limitations. Since it was a cross-sectional study, a causal association could not be established between socio-demographic factors and dietary patterns. The portion size was not obtained, and hence the actual intakes of the nutrients were not calculated. FFQ methodology for dietary intake analysis is limited by its accuracy, needs a larger sample size, and has possible errors, but the use of established criteria and practices kept the biases and errors at a minimum level. We incorporated all the possible food groups, including local foods during interviews, as mentioned in the past literature or national guidelines by the Indian Council of Medical Research. Since the food group categories are broad, foods weakly associated with the dietary pattern were clubbed in the same category as the food strongly associated with that pattern. This limits the details gathered by a specific pattern and sensitivity of the components and their associations with variables.

The present study was population-based with the focus on marginalized (marginalized classes) and vulnerable populations, pregnant and lactating mothers. The response rate was nearly 100%. The collection of data from two different settings (rural and urban slums) helped researchers accumulate data on heterogeneity in their dietary patterns. There have been limited studies with such an in-depth analysis and mentioning about ICDS relations with dietary patterns of women who are the ultimate user of such services. Our study advances the evidence base on nutrition-related data of women in the Indian context. Both ICDS and antenatal care programs are at the prime focus of any nutrition-specific intervention with this segment of the population.

## Conclusions

Largely, pregnant and lactating women had low-mixed vegetarian dietary patterns, which were greatly influenced by religion and areas of residence. A vegetarian diet without fruits and pulses is poor in nutrients, and such a low-mixed diet may lead to maternal undernutrition and micronutrient deficiencies. Occupation and education status have also been found to affect dietary patterns. Nutrition-related advice during pregnancy and lactation may help women improve diet and promote the consumption of a high-mixed diet rich in fruits and proteins. Our findings suggest that visits to healthcare services (such as during antenatal or postnatal period) should be utilized as opportunities to counsel pregnant and lactating women for the consumption of mixed-diets, rich in fruits, non-vegetarian food items, eggs, and pulses. Further, women should be advised to take supplementary foods supplied under the ICDS scheme.

## Data Availability Statement

The raw data supporting the conclusions of this article will be made available by the authors, without undue reservation.

## Ethics Statement

The studies involving human participants were reviewed and approved by MAMTA Ethical Review Board (MAMTA Health Institute for Mother and Child). The patients/participants provided their written informed consent to participate in this study.

## Author Contributions

SS devised the research study with substantial guidance from SM and FA and wrote the first draft of the manuscript. All data analysis was undertaken by SS with RK supporting in data quality assurance and cleaning. SM and FA provided critical revisions and feedback. All authors contributed to the concept and design of the overall research and data interpretation.

## Conflict of Interest

The authors declare that the research was conducted in the absence of any commercial or financial relationships that could be construed as a potential conflict of interest.
